# Radiographic Identification of Cardiac Implantable Electronic Device Manufacturer: Smartphone Pacemaker-ID Application Versus X-ray Logo

**DOI:** 10.19102/icrm.2022.130803

**Published:** 2022-08-15

**Authors:** Bridget Boyle, Charles J. Love, Joseph E. Marine, Jonathan Chrispin, Andreas S. Barth, John W. Rickard, David D. Spragg, Ronald Berger, Hugh Calkins, Sunil K. Sinha

**Affiliations:** ^1^Division of Cardiology, Johns Hopkins University School of Medicine, Baltimore, MD, USA; ^2^Department of Cardiovascular Medicine, Cleveland Clinic, Cleveland, OH, USA

**Keywords:** Chest X-ray, implantable cardioverter-defibrillator, pacemaker, smartphone application

## Abstract

Radiographic identification of the cardiac implantable electronic device (CIED) manufacturer facilitates urgent interrogation of an unknown CIED. In the past, we relied on visualizing a manufacturer-specific X-ray logo. Recently, a free smartphone application (“Pacemaker-ID”) was made available. A photograph of a chest X-ray was subjected to an artificial intelligence (AI) algorithm that uses manufacturer characteristics (canister shape, battery design) for identification. We sought to externally validate the accuracy of this smartphone application as a point-of-care (POC) diagnostic tool, compare on-axis to off-axis photo accuracy, and compare it to X-ray logo visualization for manufacturer identification. We reviewed operative reports and chest X-rays in 156 pacemaker and 144 defibrillator patients to visualize X-ray logos and to test the application with 3 standard (on-axis) and 4 non-standard (off-axis) photos (20° cranial; caudal, leftward, and rightward). Contingency tables were created and chi-squared analyses (P < .05) were completed for manufacturer and CIED type. The accuracy of the application was 91.7% and 86.3% with single and serial application(s), respectively; 80.7% with off-axis photos; and helpful for all manufacturers (range, 85.4%–96.6%). Overall, the application proved superior to the X-ray logo, visualized in 56% overall (P < .0001) but varied significantly by manufacturer (range, 7.7%–94.8%; P < .00001). The accuracy of the Pacemaker-ID application is consistent with reports from its creators and superior to X-ray logo visualization. The accuracy of the application as a POC tool can be enhanced and maintained with further AI training using recent CIED models. Some manufacturers can enhance their X-ray logos by improving placement and design.

## Introduction

**Figure 1:**
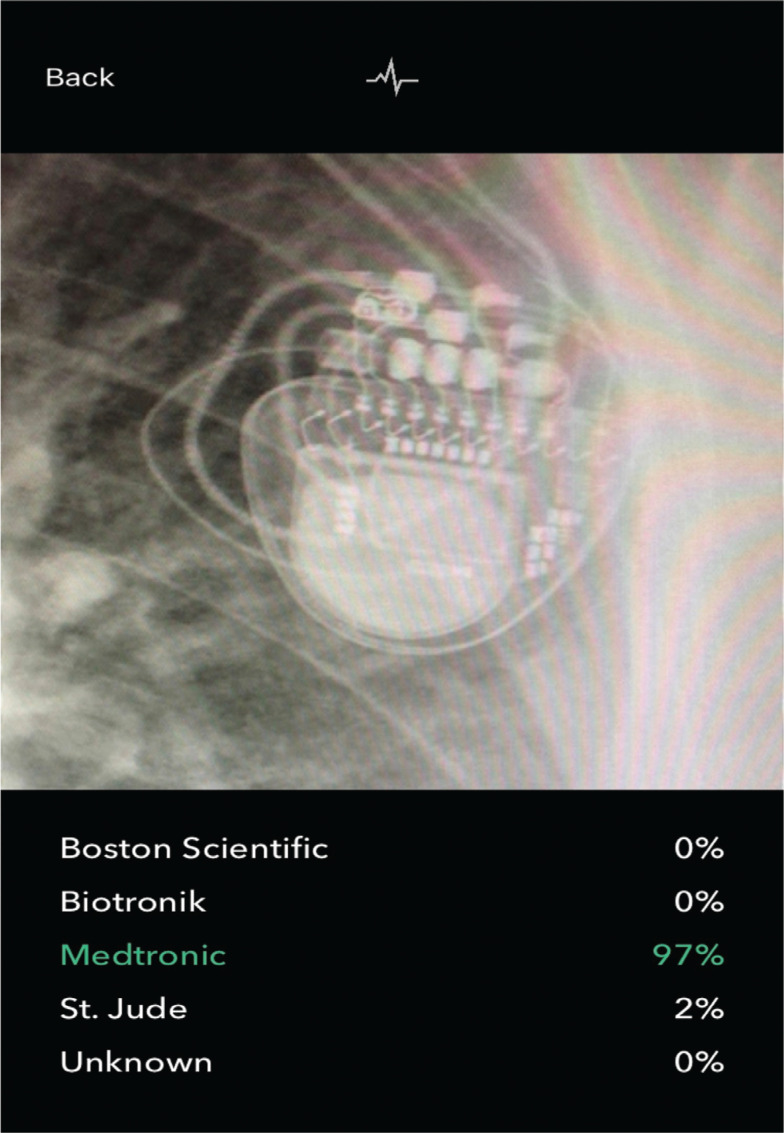
Screenshot of Pacemaker-ID application smartphone photo with automatic identification of pacemaker manufacturer expressed with percentage of certainty.

**Figure 2:**
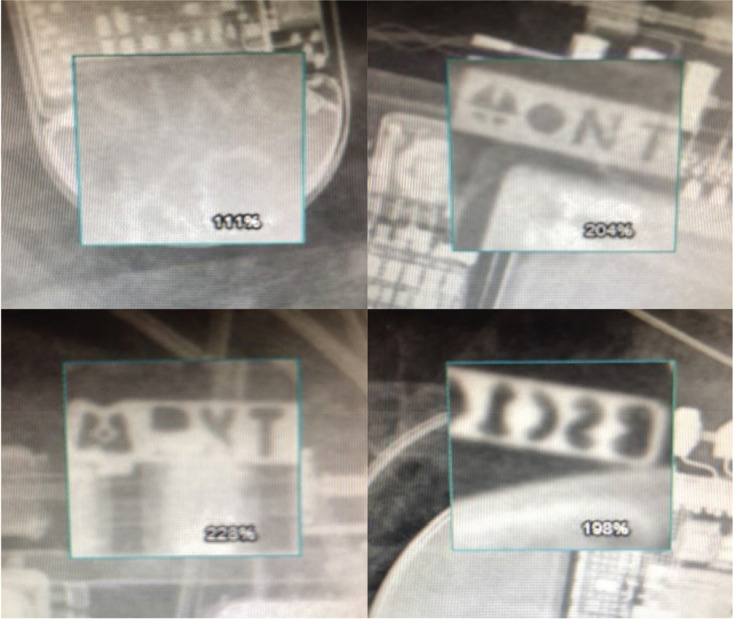
The manufacturer-specific X-ray logos from left to right in clockwise rotation from top left: Abbott, Biotronik, Boston Scientific, and Medtronic.

Prompt radiographic identification of an unknown cardiac implantable electronic device (CIED) manufacturer can facilitate urgent pacemaker or defibrillator interrogation with the appropriate manufacturer-specific computer programmer. This scenario is of clinical importance when a patient with a CIED presents urgently/emergently to a health system with cardiorespiratory symptoms but without the knowledge of their CIED manufacturer or relevant medical records to identify it. In the past, radiographic options have involved visualizing a manufacturer-specific X-ray logo or using a comprehensive but very complex chart publication of X-ray images.^1,2^ In 2019, a novel smartphone application (“Pacemaker-ID”) provided a new diagnostic approach and has been made available for free download. The Pacemaker-ID application acquires a smartphone photograph of a chest X-ray image (postero-anterior [PA] or antero-posterior [AP] view) and subjects it to an artificial intelligence (AI) algorithm that uses key characteristics (canister shape, battery design) to identify the manufacturer with the degree of certainty provided as a percentage (see **Figure 1** for a screenshot). Its creators initially reported an accuracy of 94% when validated using 300 chest X-ray images encompassing transvenous pacemakers and defibrillators from the 4 major CIED manufacturers.^3^ Further validation by its creators of this potentially helpful point-of-care (POC) diagnostic tool has indicated an accuracy of 89% when directly compared to the very complex “CaRDIA-X” radiographic chart algorithm.^4^ Additionally, although manufacturer-specific X-ray logos have been incorporated into CIED model designs for almost 4 decades (see **Figure 2** for examples from the 4 major manufacturers), a formal analysis of their diagnostic utility has never been provided.

We sought to independently assess the accuracy of the Pacemaker-ID application as a POC diagnostic tool using not only “standard” (on-axis) photos but also “non-standard” (off-axis) photos. Additionally, we sought to compare its accuracy to the diagnostic utility of the manufacturer-specific X-ray logo across all major manufacturers.

## Methods

Our retrospective cohort analysis included 319 new consecutive CIED placements with postoperative chest X-rays documented in our electronic medical record at 1 of 3 Johns Hopkins Medical Institutions—Johns Hopkins Hospital (Baltimore, MD, USA), Johns Hopkins Bayview Medical Center (Baltimore, MD, USA), and Howard County General Hospital (Columbia, MD, USA)—between January 1, 2013, and December 31, 2020. Operative reports were reviewed, and CIED characteristics (manufacturer, model, and CIED type—pacemaker or defibrillator) were cataloged once for each specific device. Postoperative chest X-ray images were reviewed by 2 investigators (B. B., S. K. S.) and cataloged by type—PA view or AP view. Alternative X-ray views, suboptimal chest X-rays (overexposed or underexposed images), and other imaging modalities (eg, computed tomography) were excluded.

### Image analysis

Each computerized chest X-ray image was systematically assessed for successful visualization of the manufacturer-specific X-ray logo and accurate determination by the Pacemaker-ID smartphone application. Specifically, a smartphone photo of the CIED was taken and each application result for manufacturer determination (>50% certainty) was cataloged on 3 consecutive attempts using a “standard” on-axis view. Additionally, a smartphone photo of the CIED was taken and each application determination for manufacturer was cataloged on consecutive attempts using 4 different “non-standard” off-axis views (20° cranial, caudal, leftward, and rightward) by manually employing a 6-in protractor with an adjustable swing arm. Computerized chest X-ray image enhancement tools such as “magnification” and/or “contrast adjustment” were permitted for optimal visualization and/or photography.

### Statistical analysis

Our pre-specified cohorts included X-ray logo visualization, single and serial (3 consecutive) “standard” on-axis smartphone photo Pacemaker-ID applications, and “non-standard” off-axis smartphone photo Pacemaker-ID applications, grouped by CIED manufacturer, CIED type (pacemaker or defibrillator), and chest X-ray type (PA or AP view). An ad-hoc subgroup analysis was also performed for cardiac resynchronization therapy (CRT) pacemakers versus CRT defibrillators. Contingency tables were composed for categorical variables and compared using chi-squared analysis with application of the Yates correction when appropriate. A 2-tailed *P* value of < .05 was considered to be statistically significant.

This was an observational study. The study protocol was approved by the institutional review boards of the Johns Hopkins University School of Medicine (IRB00253112) and Howard County General Hospital.

## Results

A total of 319 operative reports and 304 computerized chest X-ray images were reviewed. Four were excluded due to suboptimal image quality (marked underexposure). An additional 15 were excluded because they were non-transvenous CIEDs (10 Medtronic Micra leadless pacemakers, 4 Medtronic Reveal Linq monitors, and 1 Abbott Confirm monitor) that lacked manufacturer-specific X-ray logos and were not included in the Pacemaker-ID application’s training set repertoire of transvenous pacemakers and defibrillators. The primary analysis included 300 CIEDs: 156 pacemakers and 144 defibrillators encompassing 4 major manufacturers—Abbott (Chicago, IL, USA), n = 104; Biotronik (Berlin, Germany), n = 41; Boston Scientific (formerly Guidant, Marlborough, MA, USA), n = 59; and Medtronic (Minneapolis, MN, USA), n = 96. The 300 associated postoperative chest X-rays available for review included 246 PA views and 54 AP views.

### X-ray logo analysis

**Figure 3:**
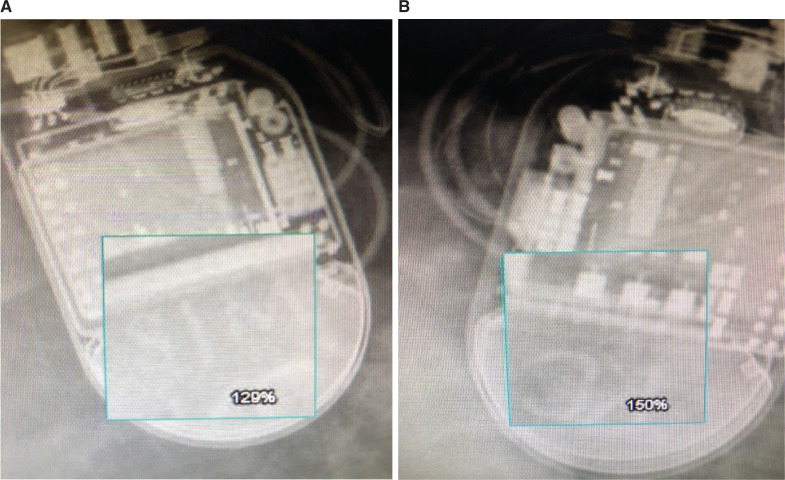
**A:** The X-ray logo of an Abbott (St. Jude Medical) defibrillator is visible although faint in front-to-back orientation. **B:** The X-ray logo of a similar Abbott (St. Jude Medical) defibrillator model is not visible in a tilted back-to-front orientation.

The manufacturer-specific X-ray logo was visualized in 56% (168/300) of X-rays overall and was seen similarly in pacemakers (54.5%) and defibrillators (57.6%) (see **Table 1**). However, there was significant variation in visualization of the X-ray logo amongst manufacturers ranging from only 7.7% and 43.9% for Abbott and Biotronik, respectively, up to 86.4% and 94.8% for Boston Scientific and Medtronic, respectively (*P* < .00001). In particular, we noted that the X-ray logo in recent Abbott CIED models was extremely faint and difficult to ascertain from its surroundings, while the X-ray logo in many Biotronik CIED models could not be discerned when it overlapped with adjacent radio-opaque components in the pulse generator (see **Figures 3A and 3B** for examples of faint and non-discernible X-ray logos). Conversely, we noted that the Medtronic X-ray logo design and placement allowed for it to be readily visualized in all models regardless of orientation and angle of the CIED imaged **(Figure 2)**. Overall, there was a significant difference in visualizing the X-ray logo based upon chest X-ray type (PA view vs. AP view, 60.2% vs. 37.0%; *P* < .001) (see **Table 2**).

### Pacemaker-ID application analysis

Overall, the Pacemaker-ID application, using “standard” on-axis smartphone photos, yielded an accuracy of 91.7% (275/300) on single photo acquisition with a similar serial accuracy of 86.3% (259/300), whereupon the application’s determinations proved congruent on 3 consecutive photo acquisitions (91.7% vs. 86.3%, *P* = not significant [NS]). This approach proved significantly higher than the visualization of the X-ray logo for the same set of chest X-rays (91.7% vs. 56%, *P* < .0001) (see **Table 1**). The serial accuracy of the Pacemaker-ID application was significantly lower in pacemakers (78.8%) compared to defibrillators (94.4%) (*P* < .0001). This discrepancy was, in part, attributable to its reduced accuracy in a subset of 19 Medtronic-manufactured CRT-pacemakers as compared to 26 Medtronic-manufactured CRT-defibrillators (68.4% vs. 92.3%, respectively).

In contrast to X-ray logo visualization, there was no significant difference in accuracy when comparing the Pacemaker-ID application’s performance based upon chest X-ray type (PA view vs. AP view, 88.2% vs. 77.8%; *P* = NS) (see **Table 3**).

Finally, we assessed the accuracy of the Pacemaker-ID application when smartphone photos were acquired in 4 different “non-standard” off-axis angles and found no significant difference between 20° cranial, caudal, leftward, and rightward angles (range, 88.7%–90.3%; *P* = NS) (see **Table 4**). In fact, the overall accuracy (4/4) of all 4 off-axis photos was 80.7%, which proved to be an insignificant decrease from the 86.3% accuracy seen with serial on-axis photos (*P* = NS) (see **Table 1**).

## Discussion

Prompt CIED interrogation is usually warranted when a pacemaker or defibrillator patient presents urgently/emergently with new-onset heart failure, palpitations, syncope, and/or defibrillator shocks. However, the facilitation of either bedside or remote interrogation requires accurate identification of the CIED manufacturer for use of the appropriate manufacturer-specific programmer. When identification is not forthcoming from the available electronic medical records or the patient’s recollection (eg, possession of a manufacturer ID card), a chest X-ray, which is often included as part of a patient’s initial cardiac work-up, can facilitate prompt identification.^1^ In this regard, we sought to independently validate and comprehensively evaluate the recently released Pacemaker-ID smartphone application as a POC diagnostic tool in several different circumstances. We observed that the diagnostic accuracy of Pacemaker-ID application was 91.7% when validated in 300 CIED X-ray images and thus comparable with the initial (94%) and subsequent (89%) accuracy estimates provided by its creators.^3,4^ Additionally, the diagnostic accuracy of this application remained 86.3% with serial use on 3 consecutive photo acquisitions confirming its reproducibility and dropped only modestly to 80.7% when photos were acquired in a “non-standard” off-axis manner intended to emulate suboptimal “real-world” use.

Furthermore, we demonstrated that the Pacemaker-ID application was superior to the diagnostic utility of the longstanding option of X-ray logo visualization (91.7% vs. 56%, *P* < .0001). The latter proved dependent on the type of X-ray view (60.2% in PA vs. 37% in AP, *P* < .001), although significant variance among specific manufacturers was noted as summarized in the following.

### Abbott

A total of 104 CIEDs manufactured by Abbott were analyzed, including 56 pacemakers and 48 defibrillators. The Pacemaker-ID application’s diagnostic accuracy of 96.2% and 88.5% on single and serial photo acquisition(s), respectively, proved far superior to the X-ray logo visualization rate of only 7.7% (*P* < .0001). We noted that almost all recent pacemaker and defibrillator models for this manufacturer lacked a clearly discernible X-ray logo.

### Biotronik

A total of 41 CIEDs manufactured by Biotronik were analyzed, including 21 pacemakers and 20 defibrillators. The Pacemaker-ID application’s diagnostic accuracy of 85.4% and 75.6% on single and serial photo acquisition(s), respectively, proved superior to the X-ray logo visualization rate of 43.9% (*P* < .01). We noted that many pacemaker and defibrillator models for this manufacturer had an X-ray logo that was difficult to discern when the pulse generator was positioned back to front or at an angle resulting in overlap with adjacent radio-opaque components.

### Boston Scientific

A total of 59 CIEDs manufactured by Boston Scientific were analyzed, including 31 pacemakers and 28 defibrillators. The Pacemaker-ID application’s diagnostic accuracy of 96.6% on both single and serial photo acquisition(s) was not significantly different from the X-ray logo visualization rate of 86.4% for this manufacturer (*P* = NS). However, X-ray logo visualization was significantly higher in the PA view (95.7%) compared to the AP view (53.8%) (*P* < .001).

### Medtronic

A total of 96 CIEDs manufactured by Medtronic were analyzed, including 48 pacemakers and 48 defibrillators. The Pacemaker-ID application’s diagnostic accuracy of 86.5% and 82.3% on single and serial photo acquisition(s), respectively, was significantly lower than the X-ray logo visualization rate of 94.8% for this manufacturer (*P* < .02). In particular, we noted that the Medtronic X-ray logo design and placement allowed for it to be readily visualized in all models regardless of X-ray view, orientation, or angle of the CIED imaged.

### Study limitations

The most significant limitation to the reproducibility of our study results is the heterogeneity of our patient population in regard to the manufacturer-specific CIED models analyzed. We endeavored to use the same 4 large manufacturers included in original reports of the Pacemaker-ID application. Nevertheless, the accuracy provided by this application could vary depending on the local distribution of manufacturer-specific CIED models. For example, we noted lower recognition of Medtronic CRT pacemakers commonly implanted at our institutions.

Additionally, as both reviewers (B. B., S. K. S.) are experienced health care professionals who often review chest X-rays in clinical practice, and were not blinded to CIED manufacturer, we cannot rule out potential selection bias when manipulating chest X-ray images and/or visualizing X-ray logos.

## Conclusion

We independently validated the accuracy of the Pacemaker-ID application to be consistent with reports from its creators and superior to X-ray logo visualization even when used in a suboptimal manner. The application’s accuracy can likely be enhanced and maintained with ongoing AI data training sets utilizing the most recent CIED models. Some manufacturers can enhance their X-ray logo’s visualization by improving logo placement and design.

## Figures and Tables

**Table 1:** Cardiac Implantable Electronic Device Manufacturer Identification Rates and X-ray Logo Versus Pacemaker-ID Application On-axis ×1, On-axis ×3, and On-axis Versus Off-axis Accuracy

Manufacturer Identification: X-ray Logo vs. Smartphone “Pacemaker-ID” App						
Manufacturer (n = CIEDs)	Manufacturer-specific X-ray Logo	Pacemaker-ID App ×1 (On-axis)	Pacemaker-ID App ×3 (On-axis)	Pacemaker-ID App ×4 (Off-axis)	X-ray Logo vs. On-axis	On-axis ×3 vs. Off-axis ×4
**Abbott (104)**	**7.7% (8/104)**	**96.2% (100/104)**	**88.5% (92/104)**	**84.6% (88/104)**	***P* < .0001**	*P* = NS
Pacemaker	3.6% (2/56)	94.6% (53/56)	83.9% (47/56)	80.4% (45/56)	***P* < .0001**	*P* = NS
Defibrillator	12.5% (6/48)	97.9% (47/48)	93.8% (45/48)	89.6% (43/48)	***P* < .0001**	*P* = NS
**Biotronik (41)**	**43.9% (18/41)**	**85.4% (35/41)**	**75.6% (31/41)**	**65.9% (27/41)**	***P* < .01**	*P* = NS
Pacemaker	47.6% (10/21)	76.2% (16/21)	57.1% (12/21)	47.6% (10/21)	*P* = NS	*P* = NS
Defibrillator	40.0% (8/20)	95.0% (19/20)	95.0% (19/20)	85.0% (17/20)	***P* < .001**	*P* = NS
**Boston Scientific (59)**	**86.4% (51/59)**	**96.6% (57/59)**	**96.6% (57/59)**	**88.1% (52/59)**	*P* = NS	*P* = NS
Pacemaker	80.6% (25/31)	100% (31/31)	100% (31/31)	96.8% (30/31)	***P* < .05**	*P* = NS
Defibrillator	92.9% (26/28)	92.9% (26/28)	92.9% (26/28)	78.6% (22/28)	*P* = NS	*P* = NS
**Medtronic (96)**	**94.8% (91/96)**	**86.5% (83/96)**	**82.3% (79/96)**	**78.1% (75/96)**	*P* < .02*	*P* = NS
Pacemaker	100% (48/48)	77.1% (37/48)	68.8% (33/48)	60.4% (29/48)	*P* < .001*	*P* = NS
Defibrillator	89.6% (43/48)	95.8% (46/48)	95.8% (46/48)	95.8% (46/48)	*P* = NS	*P* = NS
**Total CIEDs (300)**	**56.0% (168/300)**	**91.7% (275/300)**	**86.3% (259/300)**	**80.7% (242/300)**	***P* < .0001**	*P* = NS
**Pacemakers**	**54.5% (85/156)**	**87.8% (137/156)**	**78.8% (123/156)**	**73.1% (114/156)**	***P* < .0001**	*P* = NS
**Defibrillators**	**57.6% (83/144)**	**95.8% (138/144)**	**94.4% (136/144)**	**88.9% (128/144)**	***P* < .0001**	*P* = NS

*Abbreviations:* CIED, cardiac implantable electronic device; NS, not significant. *The X-ray logo was superior to the Pacemaker-ID app for CIED identification in these specific subgroups.

**Table 2:** Cardiac Implantable Electronic Device Manufacturer Identification and Secondary Analysis of Manufacturer-specific X-ray Logo Comparing Postero-anterior and Antero-posterior X-ray Views

Manufacturer Identification: Manufacturer X-ray Logo Secondary Analysis			
Manufacturer (n = CIEDs)	X-ray Logo PA View Accuracy	X-ray Logo AP View Accuracy	X-ray Logo PA View vs. AP View*
**Abbott (104)**	**9.5% (8/84)**	**0.0% (0/20)**	***P* = NS**
Pacemaker	4.8% (2/42)	0.0% (0/14)	N/A
Defibrillator	14.3% (6/42)	0.0% (0/6)	N/A
**Biotronik (41)**	**46.9% (15/32)**	**33.3% (3/9)**	***P* = NS**
Pacemaker	44.4% (8/18)	66.7% (2/3)	N/A
Defibrillator	50.0% (7/14)	16.7% (1/6)	N/A
**Boston Scientific (59)**	**95.7% (44/46)**	**53.8% (7/13)**	***P <* .001**
Pacemaker	92.0% (23/25)	33.3% (2/6)	N/A
Defibrillator	100% (21/21)	71.4% (5/7)	N/A
**Medtronic (96)**	**96.4% (81/84)**	**83.3% (10/12)**	***P* = NS**
Pacemaker	100% (43/43)	100% (5/5)	N/A
Defibrillator	92.7% (38/41)	71.4% (5/7)	N/A
**Total CIEDs (300)**	**60.2% (148/246)**	**37.0% (20/54)**	***P* < .001**
**Pacemakers**	**59.4% (76/128)**	**32.1% (9/28)**	***P* < .025**
**Defibrillators**	**61.0% (72/118)**	**42.3% (11/26)**	***P* = NS**

*Abbreviations:* AP, antero–posterior; CIED, cardiac implantable electronic device; NS, not significant; PA, postero–anterior. *N/A means statistical analysis not performed due to insufficient numbers in ≥1 groups.

**Table 3:** CIED Manufacturer Identification and Secondary Analysis of Pacemaker-ID Application with On-axis Photos Comparing Postero-anterior and Antero-posterior X-ray Views

Manufacturer Identification: Smartphone Pacemaker-ID Secondary Analysis			
Manufacturer (n = CIEDs)	Pacemaker-ID PA View (On-axis) Accuracy	Pacemaker-ID AP View (On-axis) Accuracy	Pacemaker-ID PA View vs. AP View*
**Abbott (104)**	**91.7% (77/84)**	**75.0% (15/20)**	***P* = NS**
Pacemaker	85.7% (36/42)	78.6% (11/14)	*P* = NS
Defibrillator	97.6% (41/42)	66.7% (4/6)	N/A
**Biotronik (41)**	**75.0% (24/32)**	**77.8% (7/9)**	***P* = NS**
Pacemaker	61.1% (11/18)	33.3% (1/3)	N/A
Defibrillator	92.9% (13/14)	100% (6/6)	N/A
**Boston Scientific (59)**	**100% (46/46)**	**84.6% (11/13)**	***P* = NS**
Pacemaker	100% (25/25)	100% (6/6)	N/A
Defibrillator	100% (21/21)	71.4% (5/7)	N/A
**Medtronic (96)**	**83.3% (70/84)**	**75.0% (9/12)**	***P* = NS**
Pacemaker	69.8% (30/43)	60.0% (3/5)	N/A
Defibrillator	97.6% (40/41)	85.7% (6/7)	N/A
**Total CIEDs (300)**	**88.2% (217/246)**	**77.8% (42/54)**	***P* = NS**
**Pacemakers**	**79.7% (102/128)**	**75.0% (21/28)**	***P* = NS**
**Defibrillators**	**97.5% (115/118)**	**80.8% (21/26)**	***P* < .005**

*Abbreviations:* AP, antero-posterior; CIED, cardiac implantable electronic device; NS, not significant; PA, postero-anterior. *N/A means statistical analysis not performed due to insufficient numbers in ≥1 groups.

**Table 4:** Cardiac Implantable Electronic Device Manufacturer Identification and 4 Distinct “Non-standard” Off-axis Photo Angles Assessed and Summated for Cumulative Accuracy

Manufacturer Identification: Off-axis “Pacemaker-ID” Analysis					
Manufacturer (n = CIEDs)	20° Caudal	20° Cranial	20° Leftward	20° Rightward	Pacemaker-ID Off-axis Accuracy
**Abbott (104)**	**90.4% (94/104)**	**95.2% (99/104)**	**95.2% (99/104)**	**91.3% (95/104)**	**84.6% (88/104)**
Pacemaker	87.5% (49/56)	96.4% (54/56)	91.1% (51/56)	85.7% (48/56)	80.4% (45/56)
Defibrillator	93.8% (45/48)	93.8% (45/48)	100% (48/48)	97.9% (47/48)	89.6% (43/48)
**Biotronik (41)**	**73.2% (30/41)**	**78.0% (32/41)**	**85.4% (35/41)**	**82.9% (34/41)**	**65.9% (27/41)**
Pacemaker	57.1% (12/21)	71.4% (15/21)	76.2% (16/21)	71.4% (15/21)	47.6% (10/21)
Defibrillator	90% (18/20)	85% (17/20)	95% (19/20)	95% (19/20)	85.0% (17/20)
**Boston Scientific (59)**	**94.9% (56/59)**	**93.2% (55/59)**	**94.9% (56/59)**	**96.6% (57/59)**	**88.1% (52/59)**
Pacemaker	100% (31/31)	100% (31/31)	96.8% (30/31)	100% (31/31)	96.8% (30/31)
Defibrillator	(25/28)	(24/28)	(26/28)	(26/28)	78.6% (22/28)
**Medtronic (96)**	**89.6% (86/96)**	**89.6% (86/96)**	**87.5% (84/96)**	**88.5% (85/96)**	**78.1% (75/96)**
Pacemaker	81.3% (39/48)	79.2% (38/48)	79.2% (38/48)	79.2% (38/48)	60.4% (29/48)
Defibrillator	97.9% (47/48)	100% (48/48)	95.8% (46/48)	97.9% (47/48)	95.8% (46/48)
**Total CIEDs (300)**	**88.7% (266/300)**	**90.7% (272/300)**	**91.3% (274/300)**	**90.3% (271/300)**	**80.7% (242/300)**
**Pacemakers**	**84.0% (131/156)**	**88.5% (138/156)**	**86.5% (135/156)**	**84.6% (132/156)**	**73.1% (114/156)**
**Defibrillators**	**93.8% (135/144)**	**93.1% (134/144)**	**96.5% (139/144)**	**96.5% (139/144)**	**88.9% (128/144)**

*Abbreviation:* CIED, cardiac implantable electronic device.
